# A Symptomatic Spinal Extradural Arachnoid Cyst with Lumbar Disc Herniation

**DOI:** 10.1155/2015/250710

**Published:** 2015-03-11

**Authors:** Yoshinori Kadono, Takamichi Yuguchi, Yu-ichiro Ohnishi, Koichi Iwatsuki, Toshiki Yoshimine

**Affiliations:** ^1^Department of Neurosurgery, Osaka University School of Medicine, Osaka 565-0871, Japan; ^2^Yuguchi Neuro and Spine Surgery, Osaka 543-0014, Japan

## Abstract

Spinal epidural arachnoid cyst (EAC) is a rare, usually asymptomatic condition of unknown origin, which typically involves the lower thoracic spine. We report a case of posttraumatic symptomatic EAC with lumbar disc herniation. A 22-year-old man experienced back pain and sciatica after a traffic accident. Neurological examination revealed a right L5 radiculopathy. Magnetic resonance imaging demonstrated a cystic lesion at the L3 to L5 level and an L4-5 disc herniation; computed tomography myelography showed that the right L5 root was sandwiched between the cyst and the herniation. A dural defect was identified during surgery. The cyst was excised completely and the defect was repaired. A herniation was excised beside the dural sac. Histology showed that the cyst wall consisted of collagen and meningothelial cells. Postoperatively the symptoms resolved. Lumbar spinal EACs are rare; such cysts may arise from a congenital dural crack and grow gradually. The 6 cases of symptomatic lumbar EAC reported in the literature were not associated with disc herniation or trauma. In this case, the comorbid disc herniation was involved in symptom progression. Although many EACs are asymptomatic, comorbid spinal disorders such as disc herniation or trauma can result in symptom progression.

## 1. Introduction

Spinal epidural arachnoid cyst (EAC) accounts for approximately 1% of all primary spinal space-occupying lesions [1], is most common in young men, and usually involves the lower thoracic spine [2]. A spinal EAC is generally asymptomatic and is often an incidental finding on radiographic evaluation of the spine. Magnetic resonance imaging (MRI) is the diagnostic method of choice. MRI can noninvasively demonstrate cyst size and the anatomic relationship with the spine but is less useful in the detection of dural defects. Complete removal of the cyst and repair of the dural defect are the primary treatments for symptomatic spinal EAC [2, 3]. The etiology and pathogenesis of spinal EAC remain unclear; EACs may develop from a protrusion of the arachnoid, through a small crack or congenital defect in the dura. Subsequent enlargement due to a one-way valve can cause symptoms [4].We report a case of a symptomatic spinal EAC of probable traumatic origin, with associated lumbar disc herniation, also due to trauma.

## 2. Case Presentation

A 22-year-old man who had developed back pain and right-sided sciatica over the 1 year following a traffic accident was referred to our hospital. Shortly after the accident he experienced only back pain; sciatica gradually developed, resulting in paresthesia and gait disturbance. Neurological examination revealed right L5 radiculopathy; the straight leg raise test was positive in his right foot at 50°. Deep tendon reflexes were preserved and there were no rectal or bladder symptoms. His lumbar severity score, according to the Japan Orthopedic Association (JOA) scoring system, was 15. Routine laboratory test results were normal. The patient's medical history was unremarkable, aside from 2 previous motorbike accidents, 1 year and 5 years before admission.

**Figure 1:**
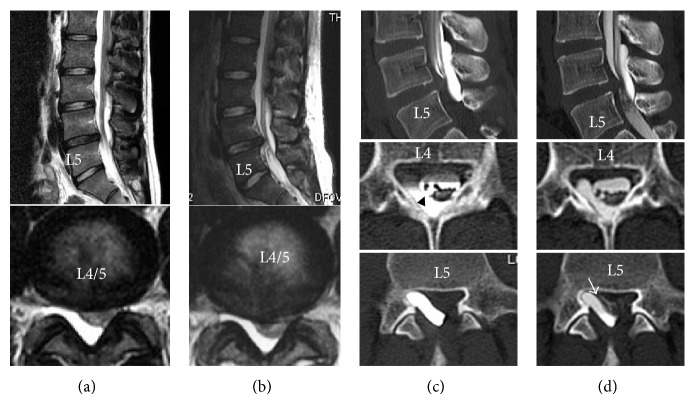
Magnetic resonance (MR) images immediately after injury show a dorsally located cystic lesion extending from L3 to L5 and disc herniation at the L4-L5 level (a). Preoperative magnetic resonance images show progression of herniation and compression of the dural sac (b). Computerized tomography (CT) myelogram shows the communication site at the L4 level ((c), arrowhead). Compression of the cauda equina and blockage of CSF at the L4-L5 level are also shown. CT myelogram obtained 2 h later demonstrates the L5 root sandwiched between the cyst and herniation ((d), arrow).

A lumbar roentgenogram revealed almost normal alignment and no instability. Computed tomography (CT) findings were normal. MRI revealed a cystic lesion extending from L3 to the L5 body level and an L4-5 medial disc herniation. The cystic lesion was hypointense on T1-weighted images and hyperintense on T2-weighted images. A dural sac and the L5 root were compressed between the cyst and the herniation (Figure 1(a)). These 2 lesions were detected on MRI just after the trauma but had increased in size over 1 year (Figure 1(b)). We attempted conservative (bed rest) and medical management for less than 3 months, without success. A CT myelogram showed that the cyst communicated with the arachnoid space at the L4 level and contained no nerve root. The fistula was presumed to be at the upper part of the cyst. Compression of the cauda equina and blockage of cerebrospinal fluid (CSF) at the L4-L5 level were also observed (Figure 1(c)). Repeated CT myelography demonstrated that the cyst filled with contrast, while washout was delayed (Figure 1(d)), suggesting communication without a one-way valve. The patient was considered a surgical candidate.

**Figure 2:**
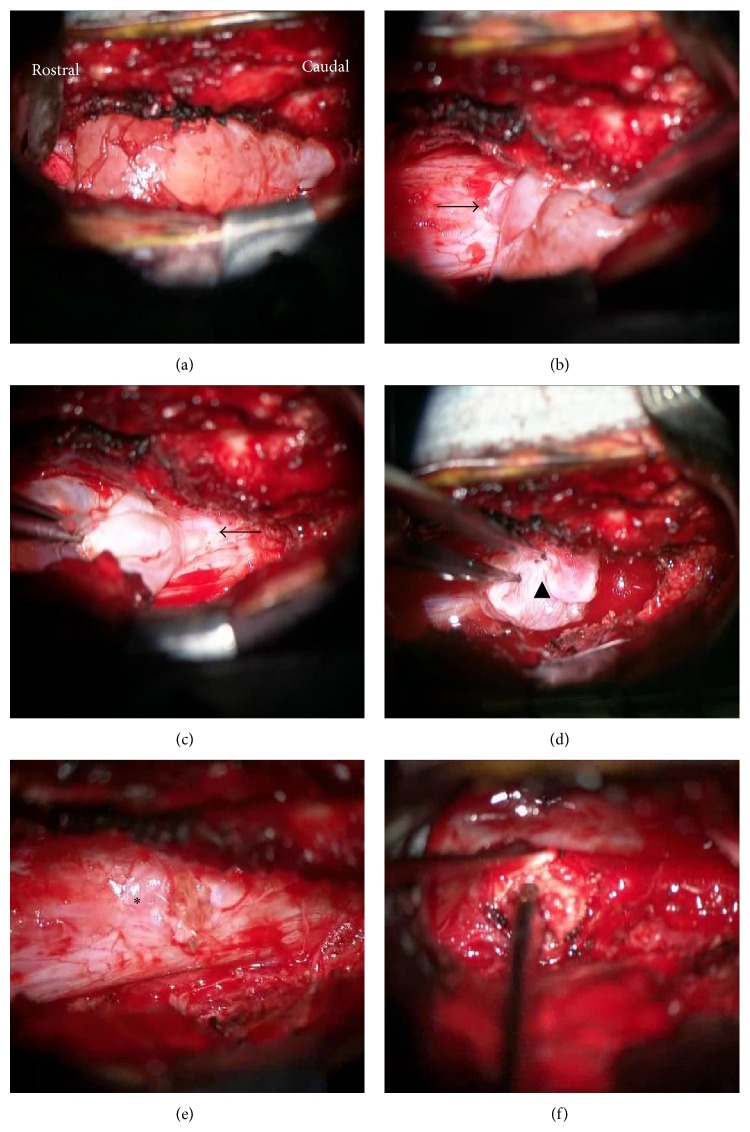
Intraoperative photographs. A right hemilaminectomy was performed and the epidural arachnoid cyst (EAC) was exposed (a). There is a small dural defect near the middle of the posterior wall ((b), (c) arrows). Puncturing the cyst, the site of communication (valveless) was seen ((d), arrowhead). The cyst wall was completely removed and the dural defect was closed by primary suture ((e), asterisk right). The disc herniation was excised from the right side (f).

**Figure 3:**
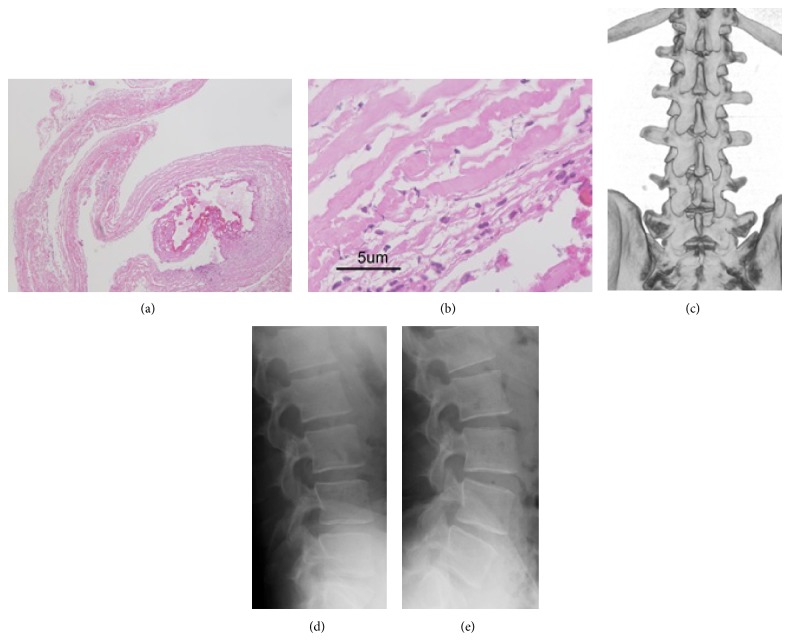
A section of the cyst wall ((a): hematoxylin and eosin, original magnification ×40 and (b): ×400) demonstrates thick collagenous fibers and some clusters of meningothelial cells with arachnoid dura within the cyst. Postoperative 3-dimensional CT (c) shows the right-side L4 hemilaminectomy and upper L5 laminotomy, with preservation of the facet joints. Dynamic plain radiography ((d), (e)) shows no instability or kyphotic deformity.

Intraoperatively, a paramedian linear incision was made over the L3–L5 spinous processes. Using microscopy, an L4 hemilaminectomy and partial laminectomy of the lower L3 and upper L5 were performed, to dissect between the cyst wall and dura mater and to expose the fistula. The dural defect was identified at the L4 level in the dorsolateral dural sac (Figures 2(b) and 2(c)). The cyst expanded with respiration. The cyst was opened; it contained no nerve and the communication site had no valve (Figure 2(d)). The cyst was completely resected, and the dural crack was repaired by primary suture. The L4-L5 disc herniation was also excised (Figure 2(f)). Histology revealed that the cyst wall consisted of collagen and some meningothelial cells, with arachnoid cells and a few lymphocytes (Figures 3(a) and 3(b)).

The postoperative course was uneventful. The sciatica improved immediately postoperatively. The JOA scores after 1 week and 6 months were 20 and 29, respectively. Postoperative CT partially revealed the drilled and resected L3–L5 laminas (Figure 3(c)). The postoperative lumbar roentgenogram revealed no change in alignment and no deterioration in instability at 6 months of followup (Figures 3(d) and 3(e)).

## 3. Discussion

Spinal EACs are a rare cause of spinal cord compression. They occur most frequently in the thoracic spine (65%) but are rare in the lumbosacral (3%) or thoracolumbar regions (3.3%) [5]. Most EACs are located posteriorly in the spinal canal [3, 6, 7]. Almost all symptomatic EACs are reported to be long or multiple and located at the thoracic or thoracolumbar level [8, 9]. We reviewed all relevant original articles and case reports published in English before July 2014, using a PubMed search. The following keywords were used in the search: symptomatic, extradural, and arachnoid cyst. Twenty-five articles were searched and 24 papers and 63 cases were reviewed. Of these, 48 reported cases at thoracic or thoracolumbar levels, and 5 reported long lesions between the thoracic and sacral levels. Four were at sacral levels, and only 6 were limited to the lumbar levels [10–14]. Retrieved data included participant characteristics, the level of the lesion, clinical symptoms, the surgical procedure, the presence of disc herniation or trauma, and the clinical outcomes (Table 1). Thoracic and cervical EACs usually present with myelopathy, while lumbar and sacral EACs are associated with lower back pain, radiculopathy, and bowel or bladder dysfunction. A few cases have been reported to extend into or through a neural foramen [10]. However, there was no description of an EAC associated with disc herniation. Symptomatic thoracic cysts are more common than symptomatic lumbar cysts, perhaps due to the narrower canal and presence of the cord. In the present case, the cyst was located in the lumbar region but was symptomatic; the comorbid disc herniation is likely to have impacted the symptoms. MRI and CT myelography revealed L5 root compression between the cyst and herniation (Figure 1(d)).

The origin of spinal EACs is uncertain; Perret et al. postulated that arachnoid cysts resulted from widening of the septum posticum, a thin membranous partition in the dorsal thoracic spinal canal [15]. Others suggest that EACs originate from a pathological proliferation and distribution of the arachnoid trabeculae during the embryonic period [16]. EACs may be associated with dural ectasia or Marfan syndrome [17], acquired factors such as inflammation or trauma, or iatrogenic factors [18]. In this case, the site of the dural crack (distant from the root sleeve) and the history of traffic accidents suggest that trauma was a probable cause. Posttraumatic symptomatic lumbar EAC has not previously been reported. Two principal hypotheses explain EAC enlargement [4, 19, 20]; a cyst may enlarge even when it does not communicate with the subarachnoid space (noncommunicating arachnoid cyst) because of fluid production by the cells of its wall; alternatively, the “ball-valve hypothesis” suggests that an anatomical communication may function as a one-way valve, allowing CSF to enter the cyst (communicating cyst) [4]. In addition, recently most investigators describe the passive fluid-transport theory to explain the cause of cyst expansion via pulsatile CSF dynamics and an osmotic gradient, with or without valve-like mechanisms [4, 21, 22]. In the early stages, pulsatile CSF dynamics may act as a factor to promote enlargement of the cyst [22]; an osmotic gradient can then facilitate further expansion [21].

In our case, neither inspection of the cyst-subarachnoid space communication nor preoperative repeated CT myelography supported the presence of a valve-like mechanism. While fluid production and an osmotic pressure gradient might be partly responsible for cyst enlargement, the interaction between the cyst and the disc herniation are likely to have accounted for symptom progression.

Although many EACs are asymptomatic, the presence of an additional spinal disease such as disc herniation or trauma can result in symptom progression.

## Figures and Tables

**Table 1:** Summary of published reports of lumbar epidural arachnoid cysts (EACs). The clinical outcomes are categorized into 3 groups: complete, good, and fair.

Author	Year	Patient	Levels	Symptoms	Approach	Cyst removal	Disc hernia	Result
Ido et al. [10]	2002	24 f	L1-2	Back pain, legs' pain	Transforaminal approach	Total	None	Complete
Chang et al. [11]	2004	12 f	L1-2, two cysts	Paraparesis	Limited laminectomy	Total	None	Complete
Durmaz et al. [12]	2009	39 m	L2-3 Lt foramen	Low back pain, radiating lt foot	L2 + L3 hemilaminectomy	Total	None	Complete
Oh et al. [13]	2012	42 m	L1-2, L3-4	Paraparesis, back pain, legs' pain	Laminoplasty	Total	None	Complete
Oh et al. [13]	2012	26 m	L1-2	Paraparesis, back pain,	Laminectomy	Total	None	Good
Tomii et al. [14]	2013	55 f	L1-2	Numbness of both legs and urinary incontinence	Laminoplasty	Total	None	Fair
